# Prior adversities predict posttraumatic stress reactions in adolescents following the Oslo Terror events 2011

**DOI:** 10.3402/ejpt.v5.23159

**Published:** 2014-05-22

**Authors:** Dag Ø. Nordanger, Kyrre Breivik, Bente Storm Haugland, Stine Lehmann, Magne Mæhle, Hanne Cecilie Braarud, Mari Hysing

**Affiliations:** 1 Regional Centre for Child and Youth Mental Health and Child Welfare, Uni Health, Uni Research, Bergen, Norway; 2 Resource Centre on Violence, Traumatic Stress and Suicide Prevention, Haukeland University Hospital, Bergen, Norway; 3 Regional Office for Children, Youth and Family Affairs, Region South, Norway; 4 Department of Social Science, Sogn of Fjordane University College, Sogndal, Norway

**Keywords:** posttraumatic stress, terror, prior trauma, adolescents

## Abstract

**Background:**

Former studies suggest that prior exposure to adverse experiences such as violence or sexual abuse increases vulnerability to posttraumatic stress reactions in victims of subsequent trauma. However, little is known about how such a history affects responses to terror in the general adolescent population.

**Objective:**

To explore the role of prior exposure to adverse experiences as risk factors for posttraumatic stress reactions to the Oslo Terror events.

**Method:**

We used data from 10,220 high school students in a large cross-sectional survey of adolescents in Norway that took place seven months after the Oslo Terror events. Prior exposure assessed was: direct exposure to violence, witnessing of violence, and unwanted sexual acts. We explored how these prior adversities interact with well-established risk factors such as proximity to the events, perceived life threat during the terror events, and gender.

**Results:**

All types of prior exposure as well as the other risk factors were associated with terror-related posttraumatic stress reactions. The effects of prior adversities were, although small, independent of adolescents’ proximity to the terror events. Among prior adversities, only the effect of direct exposure to violence was moderated by perceived life threat. Exposure to prior adversities increased the risk of posttraumatic stress reactions equally for both genders, but proximity to the terror events and perceived life threat increased the risk more in females.

**Conclusions:**

Terror events can have a more destabilizing impact on victims of prior adversities, independent of their level of exposure. The findings may be relevant to mental health workers and others providing post-trauma health care.

It has been well documented that terror and mass shootings not only cause posttraumatic problems in directly exposed individuals, but affect the surrounding population as well (Galea et al., 2003; Miguel-Tobal et al., 2006; North et al., 1999; Rubin et al., 2007; Schlenger et al., 2002). Some studies have also found markedly elevated levels of posttraumatic stress disorder (PTSD) in children and adolescents living in the surrounding areas of major critical events, such as after the Oklahoma City bombing (Pfefferbaum et al., 1999) and after 9/11 (Schuster et al., 2001).

Physical or psychological proximity to critical incidents such as terror or disaster, and perceived life threat at the time of the events, are known risk factors for PTSD in adults (Brewin, Andrews, & Valentine, 2000; Ozer, Best, Lipsey, & Weiss, 2003) and in children and adolescents (Trickey, Siddaway, Meiser-Stedman, Serpell, & Field, 2012). Also, females are commonly found to be more susceptible to PTSD than males (Olff, Langeland, Draijer, & Gersons, 2007; Ozer et al., 2003; Trickey et al., 2012). However, factors related to the exposure do not alone explain the course of post-trauma adjustment. Most people exposed to terror and disaster recover over time (McNally, Bryant, & Ehlers, 2003), in particular if they have access to social support (Bonanno, Brewin, Kaniasty, & La Greca, 2010; Brewin et al., 2000; Olff, 2012). In children and adolescents, post-event risk factors have been found to be more strongly correlated with PTSD than pre-event factors (Trickey et al., 2012).

Still, among those who develop a chronic PTSD, there may be an overrepresentation of victims of prior trauma (Breslau, Peterson, & Schultz, 2008). In the domain of terror, Bonanno, Galea, Bucciareli, and Vlahov (2007) found that two or three prior traumas increased the risk of PTSD after 9/11 among residents of inner city New York. In a study from the same area focusing on effects of childhood experiences of physical and sexual abuse, Twaite and Rodriguez-Srednicki (2004) found associations between these exposure types and PTSD and dissociative symptoms after 9/11. However, studies investigating such phenomena in the general population are rare, and, in particular, there is a lack of studies exploring how prior exposure to adversities affects responses to terror and disaster in adolescents.

In their meta-analyses of studies of risk factors for PTSD, Brewin and coworkers (2000) concluded that more research is needed because the association between prior trauma and vulnerability to subsequent traumatic exposure seems to vary among groups and populations. Therefore, the role of prior trauma needs to be explored in different populations, and prevalence figures for the studied types of prior traumas should be taken into account. In Norway, an earlier national survey of adolescents investigated exposure to and witnessing of domestic violence and sexual assaults within and outside the family (Mossige & Stefansen, 2007). Seventeen percent had been hit by a caregiver on one or more occasions, 10% had witnessed violence between caregivers on one or more occasions, and 11% had been exposed to severe sexual assault on one or more occasions. All exposure types were more frequently reported by girls.

The two successive attacks of the Oslo Terror on July 22, 2011, killed 77, of whom eight were killed by the bomb in the Governmental Quarter and 69 in the mass shootings at the Youth Wing of the Labour Party’s summer camp at Utøya, an inland island 40 kilometers west of Oslo. The terror was defined as a national disaster and made a deep impression on the entire Norwegian population (Nordanger et al., 2013; Thoresen, Aakvaag, Wentzel-Larsen, Dyb, & Hjemdal, 2012).

In an earlier study (Nordanger et al., 2013), we showed that one out of five Norwegian high school students reported physical or psychological proximity to the Oslo Terror events. Around 11% experienced substantial life threat at the time of the events, while 5% reported substantial posttraumatic stress reactions seven months after the events. Female gender, proximity, and perceived life threat were salient predictors of posttraumatic stress reactions (Nordanger et al., 2013).

The main objective of the present study was to explore the role of prior exposure to adverse experiences as a risk factor for posttraumatic stress reactions to the Oslo Terror events. More specifically we aimed to: (1) examine the prevalence of prior direct exposure to violence, witnessing of violence, and unwanted sexual acts in a representative sample of Norwegian adolescents; (2) explore the role of these three types of prior adverse experiences as predictors for posttraumatic stress reactions related to the Oslo Terror; and (3) investigate the relationship between the three types of prior adverse experiences, proximity to the terror events, perceived life threat at the time of the events, and gender as risk factors for posttraumatic stress reaction related to the Oslo Terror. Because PTSD is more prevalent among females, we also wanted to explore how gender affected the investigated associations.

Based on previous research, we hypothesized that prior direct exposure to violence, witnessing of violence and unwanted sexual acts would predict higher levels of posttraumatic stress reactions. We also hypothesized that such effects would be moderated by proximity to the terror events and by perceived life threat, meaning that adolescents with a history of adverse experiences would display more posttraumatic stress reactions the higher they scored on proximity and perceived life threat.

## Method

### Participants and procedure

The study was based on data from the “ung@hordaland study,” a large cross-sectional survey of adolescents’ mental health comprising adolescents attending high school in Hordaland County in the spring of 2012. Hordaland County is situated on the western coast of Norway around 500 km west of Oslo, with its almost 500,000 inhabitants distributed among socioeconomic groups and among urban and rural areas in a way that mirrors Norway as a whole. Data were collected seven months after the Oslo Terror, using a web-based questionnaire covering a broad range of mental health and daily life functioning issues, known risk and protective factors, and demographic background variables. UNI Health and Hordaland County Council collaborated in conducting the study.

The present study was limited to the core group of high school students born between 1993 and 1995, comprising a total invited sample of 19,121. Of these, 10,220 completed the questionnaire (or parts of it), implying a response rate of 54%. Mean age was 16.9 years (SD: 0.87). Fifty-three percent of the respondents were females, and 5.9% were defined as immigrants because both parents were born outside Norway. For more details about the sample and procedures, see Nordanger et al. (2013).

The research protocol for the study was approved by the Regional Committee for Medical Research Ethics in western Norway.

### Measures

The current study was based on two sections of the questionnaire: One addressing *adverse life experiences* and the other addressing *exposure and reactions to the Oslo Terror*.

The adverse life experiences section was introduced by the question “Have you ever experienced any of the following?” and included the following three items:

1. “Violence from an adult person (you were beaten, pulled by your hair, or the like)”
2. “Witnessed someone you know being subjected to violence”
3. “Unwanted sexual acts”

Response categories for all three times were: “No, never”(0); “Yes, once”(1); “Yes, a few times”(2); and “Yes, a series of times”(3).

From the Oslo Terror section, the following two items addressed exposure—in terms of physical or psychological proximity to the events and perceived life threat.

1. Proximity: “Were you, or anyone you know, at Utøya or in the Governmental Quarter at the time of the attack?” If yes, the site was specified, and the following response categories appeared: “I was there myself”; “a family member died”; “a family member survived”; “a friend or girl-/boyfriend died”; “a friend or girl-/boyfriend survived”; and “an acquaintance survived.” In this study, only the dichotomous entry question was applied: “No”(0) or “Yes”(1).
2. Perceived life threat: “To what extent did you perceive the terror events as a threat to your own life or the lives of someone close to you?” Response categories were: “Not at all”(0); “a little”(1); “some”(2); “quite a lot”(3); and “to a large extent”(4). Similar questioning was applied by Rubin and coworkers (2007) after the 2005 London bombings.

The Oslo Terror section also included three items, derived from the UCLA PTSD Reaction Index for DSM-IV (Steinberg, Brymer, Decker, & Pynoos, 2004), which addressed posttraumatic stress reactions of reexperiencing, avoidance, and hyperarousal, respectively:

1. “Frightening thoughts, images, or sounds from the terror events pop up in my mind even if I don’t want them to.”
2. “I try to avoid talking about the terror events or to have thoughts or feelings related to them.”
3. “When something reminds me of the terror events, I get very upset, scared, or sad”. Response categories for all three items were: “Never”(0); “1–2 times per month”(1); “1–2 times per week”(2); “3–4 times per week”(3); or “Every day or almost every day”(4). The simple sum score of the three items was used as the dependent variable in the present study and the Cronbach’s α was 0.65. The full scale has been widely applied in adolescents (e.g., Roussos et al., 2005). The approved Norwegian version was translated by the Norwegian Centre for Violence and Traumatic Stress Studies. The short version is also used in a large public health survey among adolescents in mid-Norway (Ung-HUNT; NTNU, 2013)

### Data analyses

Data were analyzed with the IBM SPSS 19 for Windows using the linear regression module (SPSS Inc., Chicago, USA). Because the dependent variable, posttraumatic stress reactions, had a rather high skewness (3.21) and kurtosis (13.28), it was logarithmic transformed (LG10(x+1)) in order to produce a more normalized distribution (skewness=1.60; kurtosis=1.58). First, in a separate analysis, we tested the exclusive effects of prior direct exposure to violence, of witnessing of violence, and of unwanted sexual acts on posttraumatic stress reaction related to the Oslo Terror. Second, in a sequential regression analysis, we tested the unique effects of these prior adversities up and above the well-established risk factors of proximity, perceived life threat and gender, as well as the interaction effects between these well-established risk factors and the prior adversities. Variables representing proximity, perceived life threat, and gender were entered in the first block, while variables representing the three types of prior adversities were entered in second block. In the third block, a total of 12 two-way interaction effects were entered representing the two-way interaction effects of gender with all the remaining variables, proximity to the terror events with all the remaining variables, and perceived life threat with all the remaining variables. The amount of missing data was rather low for the variables included in the present analyses, ranging from 0.9% (gender) to 6.6% (witnessing of violence).

Because the study investigated the effects of adversities experienced *prior* to the Oslo Terror, we checked the overlap between those who reported being present at a terror site (*n=*34) and those who reported prior exposure to and witnessing of violence once or more, and, as a test, we ran the same analyses with the group reporting presence excluded. The overlap was limited, and analyses with and without the filter showed the same results.

## Results

### The prevalence of exposure to prior adversities

In total, 10.1% of the sample reported some (once or more) prior direct exposure to violence, 17.0% to witnessing of violence, and 6.0% to unwanted sexual acts. All exposure types were more frequently reported by females (direct exposure to violence: 0.21 vs. 0.14, *F=*34.86, *p<*0.001; witnessing of violence: 0.31 vs. 0.21, *F=*46.00, *p<*0.001; unwanted sexual acts: 0.13 vs. 0.03, *F=*167,29, *p<*0.001) (Table 1).

**Table 1:** Prior direct exposure to violence, witnessing of violence, and unwanted sexual acts

	Never		Once		A few times		A series of times	
				
	*n*	%	*n*	%	*n*	%	*n*	%
Direct exposure to violence (all)	8,534	89.89	405	4.27	402	4.23	153	1.61
Females	4,516	88.31	238	4.65	256	5.01	104	2.03
Males	4,018	91.74	167	3.81	146	3.33	49	1.12
Witnessing of violence (all)	7,871	82.99	875	9.23	538	5.67	200	2.11
Females	4,106	80.38	543	10.63	329	6.44	130	2.55
Males	3,765	86.04	332	7.59	209	4.78	70	1.60
Unwanted sexual acts (all)	8,926	94.00	405	4.26	118	1.24	47	0.49
Females	4,636	90.62	345	6.74	104	2.03	31	0.61
Males	4,290	97.95	60	1.37	14	.32	16	0.37

### The role of the three types of prior adversities as predictors for posttraumatic stress reactions

**Fig. 1:**
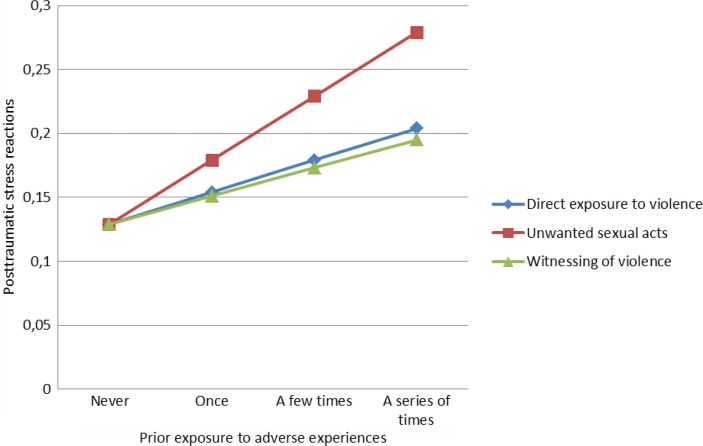
The unique effects of prior exposure to adversities on posttraumatic stress reactions (log transformed) related to the Oslo Terror (*N=*9,503).

When entered into a regression analysis, prior exposure to all of the three types of adversities contributed significantly to the prediction of the level of posttraumatic stress reactions related to the Oslo Terror (direct exposure to violence: standardized beta=0.057, *p<*0.001; unwanted sexual acts: standardized beta, 0.074, *p<*0.001; and witnessing of violence: standardized beta=0.058, *p<*0.001). As Fig. 1 illustrates, there was a steady increase in the level of posttraumatic stress reactions corresponding with the increasing number of prior exposures.

### The relative effects of prior adversities, proximity, perceived life threat and gender

Table 2 displays the unstandardized regression coefficients, associated standard errors, standardized regression coefficients (standardized beta), and t-tests for the sequential regression analyses. In Block 1, gender, proximity to the Oslo Terror and perceived life threat had a significant and independent association with posttraumatic stress reactions. In Block 2, prior direct exposure to violence, witnessing of violence and unwanted sexual acts contributed significantly to the prediction of posttraumatic stress reaction level up and above the variables entered in Block 1. However, the effect sizes were small, with standardized betas in the 0.02 to 0.04 range. The effects of gender, proximity to the terror events and perceived life threat remained almost unchanged by the inclusion of the prior exposure variables, with perceived life threat as the strongest predictor. Only three of the 12 interaction effects tested in the third block were significant (the remaining nine interaction effects were removed from the analyses). One of these involved prior exposure to adversities, showing the relationship between perceived life threat and level of posttraumatic stress reactions to be stronger the more violence to which the adolescents had previously been exposed. In addition, proximity to the terror events and perceived life threat had a stronger association with level of posttraumatic stress reactions among females compared to males.

**Table 2:** Effects of gender, proximity, perceived life threat, and prior exposure to adversities on posttraumatic stress reactions related to the Oslo Terror (*N=*9,186)

	B	SE	Stand. beta	*t*
Block 1				
Gender	−0.06	0.005	−0.12	−12.23***
Proximity to terror events	0.06	0.006	0.10	9.85***
Perceived life threat	0.08	0.002	0.33	32.99***
Block 2				
Gender	−0.06	0.005	−0.11	−11.48***
Proximity to terror events	0.06	0.006	0.09	9.25***
Perceived life threat	0.07	0.002	0.33	32.41***
Direct exposure to violence	0.02	0.004	0.04	4.00***
Unwanted sexual acts	0.02	0.007	0.03	3.42**
Witnessing of violence	0.01	0.004	0.02	2.23*
Block 3				
Gender	−0.03	0.006	−0.05	−4.38***
Proximity to terror events	0.07	0.008	0.11	8.29***
Perceived life threat	0.08	0.003	0.37	26.73***
Direct exposure to violence	0.01	0.006	0.02	1.35
Unwanted sexual acts	0.02	0.007	0.03	3.13**
Witnessing of violence	0.01	0.004	0.02	2.24*
Gender × proximity	−0.03	0.013	−0.03	−2.07*
Gender × perceived life threat	−0.03	0.005	−0.08	−5.52***
Direct exposure to violence × perceived life threat	0.01	0.003	0.03	2.36*

* *p<*0.05;

** =*p<*0.01;

*** =*p<*0.001

## Discussion

Prior direct exposure to violence, witnessing of violence, and unwanted sexual acts were predictors of posttraumatic stress reactions in adolescents following the Oslo Terror events of 2011. Overall, the strongest predictors were the known risk factors of perceived life threat, female gender, and proximity to the events, but interestingly, though the effect sizes were small, all the prior adversities had effects up and above the well-established risk factors. An exception was that perceived life threat moderated the effect of prior direct exposure to violence. Interestingly, exposure to prior adversities did not increase the risk of posttraumatic stress reactions more often in females than in men, but proximity and perceived life threat did increase the risk more often in females.

In line with the previous population survey among Norwegian adolescents (Mossige & Stefansen, 2007, see introduction), we found all prior adversities to be more frequently reported by females. However, compared to the previous study, the prevalence of prior direct exposure to violence and to unwanted sexual acts was lower. Especially when it comes to direct exposure to violence, a higher prevalence could be expected because our study did not exclusively focus on domestic violence. Findings may reflect the substantial measures that have been taken by Norwegian authorities to prevent child abuse (for an overview, see Saur, Hustad, & Heir, 2011) but can also be due to regional differences. The higher prevalence of prior witnessing of violence was as expected; by not specifying relation to perpetrators, our study may also have included the more common experience of witnessing fighting among peers.

Our study confirms former research that suggests increased vulnerability to posttraumatic stress reactions after subsequent trauma in general (Breslau et al., 2008) and to terror in particular (Bonanno et al., 2007; Twaite & Rodriguez-Srednicki, 2004) among prior trauma victims. We expected that the vulnerability connected to prior adversities would mainly be triggered by subsequent personal proximity to the terror. Because no such interaction effects were found, this hypothesis was not confirmed. However, threat and stress responses may be unrelated to actual proximity. Therefore, increased vulnerability among prior trauma victims could still be due to a sensitized stress response (including an over activation in the amygdala) in combination with weakened abilities to downregulate effect (McFarlane, 2010; Rauch, Shin, & Phelps, 2006; Shin, Rauch, & Pitman, 2006). In this perspective, the interaction effect between prior direct exposure to violence and perceived life threat makes sense; when violence has been experienced before, one may become alarmed by lower levels of stimuli intensity rather than by actual personal proximity to the threatening event. Why the effects of prior witnessing of violence and exposure to unwanted sexual acts were more direct and less moderated by perceived life threat is left to explain. For prior victims of violence, the Oslo Terror may have been a stronger reminder of the victims’ own trauma history and may therefore have been experienced as more threatening. However, this could imply that the three prior adversities increase risk for posttraumatic stress reactions through different mechanisms.

It has been hypothesized that females’ susceptibility to PTSD could be due to more childhood interpersonal traumatic experiences or a greater appraisal of threat (for an overview, see Olff et al., 2007). Our data indicate support for the first hypothesis in the sense that prior exposure to unwanted sexual acts and witnessing of violence (which both had independent effects on the level of posttraumatic stress reactions) were more frequently reported by females. At the same time, our data suggest that, when exposed, such prior adversities increase vulnerability to the same degree regardless of gender. However, in line with conclusions from Olff and colleagues’ review (Olff et al., 2007), our study directly supports the view that appraisal of threat moderates vulnerability to posttraumatic stress reactions in females.

Based on earlier literature, one would expect that prior adversities increase vulnerability more the earlier in life they take place and the closer the relation is to the perpetrator. In the case of domestic violence and sexual abuse from a caregiver at an early age when the child depends on other-regulation for handling stress, underdeveloped self- and affect regulation capacities as well as hypersensitivity to stress are commonly described consequences (Cloitre et al., 2009; Ford, 2009; Schore, 2009; Van der Kolk, 2005). To this discussion, the present study provides only indicative information through the association between number of prior exposures and level of posttraumatic stress reactions; when a series of prior exposures is reported, the more likely is an earlier age of debut.

Also, due to the limited scope of a broad population survey, relevant aspects of and nuances with regard to *exposure* to the terror events may have been missed. For example, there was massive media coverage of the Oslo Terror in the months leading up to the data collection (ScienceNordic, 2012). This exposure type has been found to increase the risk of PTSD (Saylor et al., 2003) and may well be more destabilizing for people with prior adverse experiences. Moreover, other confounders such as social support, socioeconomic status, and parental psychopathology were not included in the model. In their meta-analyses of risk factors for PTSD among children and adolescents, Trickey et al. (2012) found post-event factors to be strong predictors. Adolescents with a history of adverse experiences may more often come from families with additional life stress and less social support (Felitti et al., 1998), and they may therefore have had less access to factors contributing to post-trauma adjustment and recovery.

It should also be noted that the study design only allowed for a three-item questionnaire on posttraumatic stress reactions. Results should therefore be interpreted with caution because this only leaves us with an indication of the level of PTSD in the sample. Despite being applied in other relevant contexts (see earlier), the few items included as well as the adaption of these to the context of the Oslo Terror may have affected the properties of the scale. Further, the reports of prior adverse experiences are based on retrospective reports, and the result should be further corroborated in prospective studies. Also, the exclusive reliance on self-report in the present study could mean that the associations among the variables could, at least partly, be due to shared method variance. Finally, with a response rate of about 54% comprising mainly adolescents in school, this could limit the generalizability for adolescents who have dropped out of school. Although no data on nonresponders were available in the study, research from former waves of the ung@hordaland study suggests that nonparticipants may have more psychological problems than participants (Stormark, Heiervang, Heimann, Lundervold, & Gillberg, 2008).

### Implications

Further research is needed for more in-depth knowledge of how age when prior traumas took place and victims’ relation to perpetrators affects vulnerability to posttraumatic stress reactions following subsequent trauma such as exposure to terror. To ensure clinical relevance, future population based research on the issue should also include a more comprehensive measure of PTSD.

It is common knowledge that terror negatively affects the health of those being directly exposed. Our study suggests that a history of adverse experiences may increase vulnerability to posttraumatic stress reactions in adolescents regardless of whether they have been directly exposed to the terror events or not. The increased risk should be communicated to mental health workers, caretakers, teachers, the youth themselves, as well as to the general public—to better understand, support, and help the groups in question.
